# Machine-learning assisted swallowing assessment: a deep learning-based quality improvement tool to screen for post-stroke dysphagia

**DOI:** 10.3389/fnins.2023.1302132

**Published:** 2023-11-24

**Authors:** Rami Saab, Arjun Balachandar, Hamza Mahdi, Eptehal Nashnoush, Lucas X. Perri, Ashley L. Waldron, Alireza Sadeghian, Gordon Rubenfeld, Mark Crowley, Mark I. Boulos, Brian J. Murray, Houman Khosravani

**Affiliations:** ^1^Hurvitz Brain Sciences Program, Division of Neurology, Department of Medicine, Sunnybrook Health Sciences Centre, University of Toronto, Toronto, ON, Canada; ^2^Goodfellow-Waldron Initiative in Stroke Innovation and Recovery, Division of Neurology, Neurology Quality and Innovation Lab, University of Toronto, Toronto, ON, Canada; ^3^Department of Computer Science, Faculty of Science, Toronto Metropolitan University, Toronto, ON, Canada; ^4^Institute of Medical Science, University of Toronto, Toronto, ON, Canada; ^5^Interdepartmental Division of Critical Care, Faculty of Medicine, University of Toronto, Toronto, ON, Canada; ^6^Department of Electrical and Computer Engineering, University of Waterloo, Waterloo, ON, Canada

**Keywords:** stroke, dysphagia, machine learning, swallowing, neural technology, original research stroke, quality improvement, Artificial Intelligence

## Abstract

**Introduction:**

Post-stroke dysphagia is common and associated with significant morbidity and mortality, rendering bedside screening of significant clinical importance. Using voice as a biomarker coupled with deep learning has the potential to improve patient access to screening and mitigate the subjectivity associated with detecting voice change, a component of several validated screening protocols.

**Methods:**

In this single-center study, we developed a proof-of-concept model for automated dysphagia screening and evaluated the performance of this model on training and testing cohorts. Patients were admitted to a comprehensive stroke center, where primary English speakers could follow commands without significant aphasia and participated on a rolling basis. The primary outcome was classification either as a pass or fail equivalent using a dysphagia screening test as a label. Voice data was recorded from patients who spoke a standardized set of vowels, words, and sentences from the National Institute of Health Stroke Scale. Seventy patients were recruited and 68 were included in the analysis, with 40 in training and 28 in testing cohorts, respectively. Speech from patients was segmented into 1,579 audio clips, from which 6,655 Mel-spectrogram images were computed and used as inputs for deep-learning models (DenseNet and ConvNext, separately and together). Clip-level and participant-level swallowing status predictions were obtained through a voting method.

**Results:**

The models demonstrated clip-level dysphagia screening sensitivity of 71% and specificity of 77% (F1 = 0.73, AUC = 0.80 [95% CI: 0.78–0.82]). At the participant level, the sensitivity and specificity were 89 and 79%, respectively (F1 = 0.81, AUC = 0.91 [95% CI: 0.77–1.05]).

**Discussion:**

This study is the first to demonstrate the feasibility of applying deep learning to classify vocalizations to detect post-stroke dysphagia. Our findings suggest potential for enhancing dysphagia screening in clinical settings. https://github.com/UofTNeurology/masa-open-source.

## Introduction

Stroke is among the top three leading causes of mortality worldwide (Feigin et al., 2021). Acute stroke resulting in hospitalization is a serious health event with often life-long alteration of functional status (Singh and Hamdy, 2006). One of the most common serious complications of stroke is dysphagia, or swallowing dysfunction, which occurs in approximately 55% of acute stroke patients (Singh and Hamdy, 2006). Dysphagia places patients at increased risk of aspiration pneumonia which can be fatal, thus screening of swallowing status and/or a formal speech language pathologist assessment are commonplace as part of the admission process to stroke centers. It is important to note the distinction between screening tests and formal assessments, which are deployed either in succession or independently depending on a local stroke center protocol (Singh and Hamdy, 2006; Cohen et al., 2016). Screening tests such as the Toronto Bedside Swallowing Screening Test (TOR-BSST©) (Martino et al., 2009) can be performed by course-trained operators of varying backgrounds, whereas formal assessment of dysphagia is performed by a speech language pathologist (SLP). Screening test certification requires training time, coverage and/or resources including the cost of training and licensure. Furthermore, permission to use a particular screening tool may have its own associated resource burden. Clinical availability of SLPs may also be limited due to high case volumes and off-hours availability, often leading to reliance on screening tests. In some hospitals, lack of immediate access to an SLP to provide a swallowing assessment can result in patients receiving NG tubes as a precautionary measure even in those who would not otherwise receive such interventions for nutrition or hydration purposes. An increased risk of infection and aspiration, as well as increase in cost for patient care can manifest in these scenarios; these in addition to patient intrinsic factors such as overall health, oropharyngeal secretions, and feeding status. Inability to easily access dysphagia screening also impacts patient comfort, tolerance, and facilitation of early physiologic recovery.

In general, centers that use a screening test trigger an SLP assessment when the screening result is a failure. Despite these care pathways, many centers lack routine integration of validated screening tests or quick access to SLPs, and thus robust dysphagia screening has significant barriers. SLPs can diagnose and prescribe various diet consistencies and textures and can also, if indicated, perform a modified barium swallow and video fluoroscopic swallowing study (VFSS). This is considered a gold-standard of swallowing assessment, but it is not routinely deployed as a screening test. VFSS requires access to trained personnel, radioactive material (barium) and x-ray equipment. Validated screening tests such as the TOR-BSST© have been compared to the VFSS and have favorable characteristics from a screening test perspective (Martino et al., 2009). Moreover, even VFSS by SLP has an element of subjectivity and poor inter-rater reliability. This same issue of subjectivity permeates screening tests that rely on voice change. Furthermore, some screening tests that do not rely on voice alone, require repeated trials of oral fluid intake. These also often have a subjective component when it comes to voice change with successive intake trials and pose execution challenges. This was experienced during the COVID-19 pandemic in relation to both staffing availability but also risk of aerosol generation (Fritz et al., 2021). Voice change detected by audio alone could be used as an assistive tool for screening tools that rely on voice quality change.

Use of voice change, including those associated with sustained vowel sounds, have been used to screen for non-stroke dysphagia (Ryu et al., 2004). Further studies have demonstrated differences in extracted audio features between patients at risk of aspiration versus those not at risk even prior to swallowing (Kang et al., 2018). Additionally, the use of vocal recordings to detect pathologies has gained increasing research interest in recent years as various applications have been developed to automatically detect or monitor pathologies such as Parkinson’s disease, and cognitive impairment (Milling et al., 2022). This has opened the possibility of voice as an adjunct biomarker in dysphagia screening. Taken together, there is a quality gap, and hence a quality improvement opportunity where machine learning algorithms, can be deployed to reduce subjectivity and perform classification as part of dysphagia screening.

In this study we assessed state-of-the-art deep-learning models (ConvNext, DenseNet, and an ensemble) to screen for dysphagia using vocal samples from post-stroke inpatients. An existing commonly used dysphagia screening tool (TOR-BSST) was used to label audio clips. Models were used to classify individual audio clips from post-stroke inpatients, and individual audio clip scores were aggregated to predict participant dysphagia screening status. This deep learning approach aims to reduce subjectivity and improve access to rapid dysphagia screening.

## Methods

### Participants

A total of 70 patients were recruited from the inpatient neurovascular unit at Sunnybrook Health Sciences Center (comprehensive stroke center, Toronto, Canada). Patients were enrolled from 13 June 2022 to 19 January 2023 (epoch 1) and 24 January to 4 March 2023 (epoch 2) for training and testing datasets, respectively. Two patients, early in the study (study patients 7 and 9), had technical difficulties with their audio recordings resulting in poor audio quality during the first data collection epoch and were rejected, allowing for a total of 68 patient’s audio recordings to be used (AB, HM, 94% inter-rater agreement of good audio quality, see Supplementary Methods). All patients, as part of their routine clinical care, were assessed using the Toronto Bedside Swallowing Screening Test (TOR-BSST©), which involves baseline assessment of voice change, as well as repetitive swallows and assessment for dysphonia by a trained assessor (Martino et al., 2009). Our center uses TOR-BSST©, and among 36 other screening tests in a Cochrane review, TOR-BSST© is among the three identified best performing tests, allowing this screening tool to be used for our supervised learning model and pass or fail status to be the training label (Kiekens and Tognonato, 2022). Overall, 27 patients were designated as fails and 41 patients passed screening. Our study included 40 patients (58.9% of total) in a training cohort and 28 patients (41.1% of total) in the testing cohort. Note that enrollment was on a rolling basis, across several sampling days, and on those random days, enrollments occurred by sampling successive admissions to the stroke unit that were within 72 h of admission. All patients provided informed consent for data collection and the study was approved by the research ethics board at Sunnybrook Health Sciences Centre. Patients with recent stroke admitted to the stroke unit who could speak English, follow commands and whom did not have significant aphasia precluding participation were included. Patients who did not speak English, had a significant speech impairment (from other medical/neurologic conditions), or were medically unstable were excluded.

### Data collection

**FIGURE 1:**
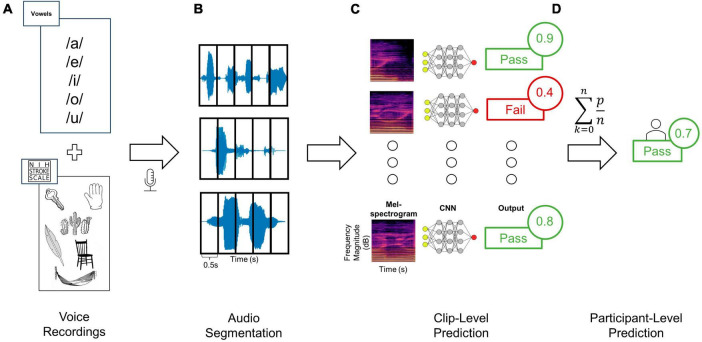
Training and testing deep learning classifiers to distinguish audio recordings based on dysphagia status. **(A)** Audio clips were recorded from each patient using a standardized assessment of vowels as well as words and sentences from the NIHSS language assessment, **(B)** and then segmented into 0.5 s windows. **(C)** Each clip from a given patient was then converted to Mel-spectrogram images using either the RGB (shown here) or three-channel approaches. Each Mel-spectrogram image was used as an input into the CNN (either DenseNet, ConvNext-Tiny or fusion networks) which generated an output class along with an output probability for each clip. **(D)** The average of all clip level output probabilities per patient were used to generate a final participant-level output class prediction.

Two categories of speech were recorded: (a) recordings of the National Institutes of Health Stroke Scale (NIHSS) portions involving speech, and (b) sustained vowel sounds (Figure 1A). The NIHSS is a widely used, validated tool for assessing neurological deficits in stroke patients, including tests of articulation, naming, repetition, and comprehension with standardized sounds, words, and sentences; we used the NIHSS to avoid bias in selecting speech tests and given its wide use in stroke assessment (Lyden et al., 1994; Appelros and Terént, 2004). In this study the NIHSS language test was separated into three distinct categories based on the type of speech: continuous speech, sentences, and words (i.e., naming objects and repeating discrete words). During segmentation these categories of speech were labeled separately for further analysis. The second category of recordings were collected by asking participants to vocalize each vowel sound (/a/, /e/, /i/, /o/, and/u/) for a target duration of 3 s repeated three times each. Prior work has demonstrated vowel sounds to be discriminative for differentiating between swallowing abnormalities and closely mirrors the articulation tasks of existing dysphagia screening tools (Waito et al., 2011; Gerratt et al., 2016; Kang et al., 2018; Moore et al., 2020). Sustained vowels were chosen since they can be more easily administered in patients for whom English is not their first language and require less vocal effort. Data was collected on an encrypted iPhone 12 with a sampling rate of 44.1 kHz using the included Voice Recorder application with a resolution of 16-bits. The phone was placed on the patient’s bedside table approximately 10 cm from their mouth. Data was collected on the inpatient stroke ward and background noise was minimized (Supplementary Methods). If there were over-head announcements during the recordings those vocalizations were asked to be repeated from the patient. The recordings were all done in a real-world setting, using an iPhone, encrypted, loss-less audio, with no other interventions to alter real-world recording conditions. This includes single and multi-patient rooms, in the ED, ward, and neurovascular step-down/observation ICU beds. Of note, investigators responsible for data collection/audio segmentation (AB, HM, LP) and the final testing phase of the model (RS) were blinded to each other’s efforts. Specifically, for most of the training data (60% of recordings during epoch 1), and for all the recordings acquired for model testing (epoch 2), the investigator running the models (RS) was blinded to the label assignment and raw audio files (see Supplementary Methods). Model training and testing was done on the spectrogram images from the audio-data (see below).

### Data analysis

Data were first assessed for quality and then analyzed through a 3-step data processing pipeline involving (1) segmentation, (2) transformation, and (3) machine learning (Figure 1). Firstly, prior to data segmentation, clips were independently evaluated for quality (Supplementary Methods). Data was segmented manually using Audacity© digital audio workstation software. Each vocalization of interest (i.e., either a vowel sound, word, sentence, or continuous speech) was segmented from the onset to the offset, labeled accordingly, and exported to an audio file. A custom data processing program was developed in Python (Supplementary Methods) to load segmented audio files, pre-process the signal, and convert them into Mel-spectrogram image representations. Given the variability in a participant’s ability to sustain vocal production for the full target duration, and large discrepancies in clip lengths with diverse sound input types (i.e., words, vowels, and continuous speech), a windowing approach was used to ensure uniform scaling of resultant Mel-spectrogram images (Zhang et al., 2019; Khurana et al., 2023). Audio clips were segmented into 0.5 s clips with 50% overlap (Figure 1B). Clips shorter than 0.5 s were rejected, and power thresholding was applied to all clip windows to ensure that periods of silence were not used to train the classifier. A single audio clip was first processed into windows. The average power contained in these windows was then calculated and windows greater than 1.5 standard deviations lower than the mean window power were rejected.

Next, all post-segmentation audio files for each participant were transformed from time-series to corresponding Mel-spectrogram images (Figure 1C). Within the realm of audio classification, the decision to employ Mel-spectrograms as opposed to directly using raw audio waveforms was both strategic and evidence-based. Mel-spectrograms are notable for their ability to emulate the human ear’s non-linear perception of pitch and frequency, making them especially powerful for tasks involving human speech (Zhang et al., 2019; Khurana et al., 2023). This is particularly salient in our study, as our ground truth anchors in evaluations by speech-language therapists. The direct mapping of these Mel-spectrograms to human auditory perception ensures that the patterns discerned by our model are grounded in clinically significant features.

**FIGURE 2:**
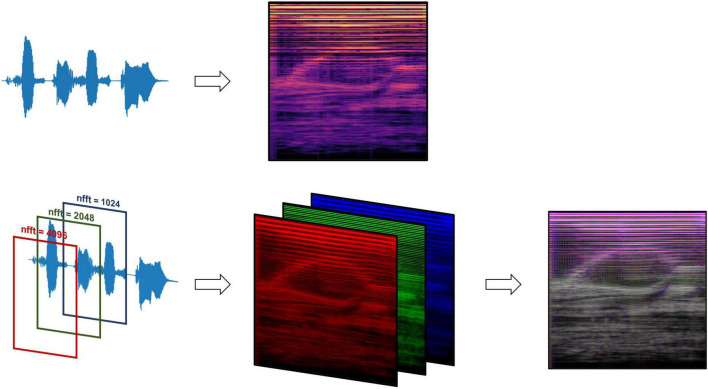
Mel-spectrogram processing methods, comparing data processing pipelines between the standard RGB Mel-spectrogram approach **(top)** and three-channel Mel-spectrogram **(bottom)** involving depth-wise concatenation of three separate Mel-spectrograms with different FFT lengths to produce a single composite image.

Mel-spectrogram images were generated using the Librosa library resulting in images with the vertical axis representing Mel frequency bands, horizontal axis representing time, and the color intensity at each point indicating the magnitude of the spectral content of that frequency and time (Schmoldt et al., 1975; Figure 2). Mel-spectrograms were computed on each clip individually with a hop length of 2,048 samples (hamming window), 512 Mels and a minimum frequency of 20 Hz. Mel-spectrograms were then converted into the power-domain (decibels) and outputted into an image file (224 × 224).

Two types of Mel-spectrogram images were generated (detailed in Supplementary Methods) to be used to train machine learning classifiers separately. The first approach (“RGB Mel-spectrogram”) used red-blue-green (RGB) images of Mel-spectrograms directly as inputs to the machine learning classifiers. The choice to use RGB Mel-spectrograms was influenced by the compatibility with transfer learning models originally trained on RGB images and the optimization of standard CNN architectures for RGB data. The second approach (“Three-channel Mel-spectrogram”) involved the depth-wise concatenations of three-monochrome Mel-spectrograms with differing Fast Fourier Transform (FFT) lengths (1,024, 2,048 and 4,096) to generate a composite image. This approach has previously demonstrated superior performance compared to RGB images in some applications (Palanisamy et al., 2020).

### Machine learning classifiers

The proposed approach relies on a type of Deep Neural Network called a convolutional neural network (CNN) which defines specialized spatial filters which allow more efficient extraction of features in images during learning (Lecun and Bengio, 1995). Extensive prior work has demonstrated the effectiveness of CNNs on other temporal and spatial data beyond images, including audio classification (Hershey et al., 2017; Zhang et al., 2019; Palanisamy et al., 2020; Dave and Srivastava, 2023; Khurana et al., 2023). CNNs were trained using transfer learning to classify Mel-spectrogram images based on TOR-BSST© screen status (Figure 1C). Transfer learning is a ML technique wherein a model is first trained on one task and then fine-tuned to solve a different task (Schmoldt et al., 1975; Hershey et al., 2017; Zhang et al., 2019; Ganaie et al., 2022; Dave and Srivastava, 2023). Large pre-trained CNN models which have been computed on large image datasets (e.g., ImageNet) have been shown to perform well in transfer learning applications when applied to images generated from audio files, even performing better than CNNs trained from scratch (i.e., with random weight initialization) (Palanisamy et al., 2020).

In this study, an ensemble method was implemented, integrating multiple classifiers trained via transfer learning, each utilizing different base models. This ensemble approach mitigates model variance stemming from random parameter initialization, thereby enhancing model robustness (Ganaie et al., 2022). Specifically, unweighted averaging was adopted for aggregating classifier outputs, a decision driven by the need for transparency and interpretability in clinical AI applications (Shortliffe and Sepúlveda, 2018). While alternative ensemble strategies, such as weighted majority voting, might offer marginal accuracy improvements by adjusting for the confidence level of individual predictions, they introduce complexities that could obscure the decision rationale (Kuncheva, 2014). In contrast, the chosen method, though simpler, maintains clarity in the decision-making process, a critical factor in healthcare settings. Importantly, a study by Ju et al. (2018) affirms the robustness of unweighted averaging, particularly when the base models exhibit similar performance levels (Ganaie et al., 2022). An ensemble network using DenseNet-121 and ConvNext-Tiny was developed. The architecture of each of these networks provides unique advantages when used as an ensemble. DenseNet uses a feed-forward connections between each layer and each subsequent layer such that a given layer N has N-1 inputs (Huang et al., 2016). This architecture allows for a lower number of parameters, and results in improved feature propagation. In applications that use computer vision for audio signals, DenseNet has demonstrated state-of-the-art (SOTA) CNN performance superior to Inception and ResNet (Palanisamy et al., 2020). Based on prior work (Palanisamy et al., 2020), all pre-trained layers were frozen until the last DenseNet block and the remaining layers in the network were fine-tuned.

ConvNext is a CNN that aims to provide some of the advantages of vision transformers. Though vision transformers have achieved SOTA performance on the ImageNet dataset in recent years they present many challenges including significant computational complexity, global rather than local attention, and reliance on large datasets (with high risk of overfitting when transfer learning is applied to smaller datasets) (Liu et al., 2022). The ConvNext includes architecture improvements that are inspired by vision transformers (ViTs) including larger kernel sizes, a processing layer (“patchify stem”) akin to a ViT patchify layer, and improved training techniques (Liu et al., 2022), resulting in classification performance similar to ViTs but with much fewer parameters. Based on training data results, all layers up to stage 3 were frozen and the remaining layers fine-tuned.

The configuration of the network architecture for both DenseNet and ConvNext is detailed in the Supplementary Methods. The last layer of each pre-trained network was removed and replaced by a global average and dense layer with a single sigmoid output. A dropout rate of 80% was used to prevent overfitting. This was settled on through empiric testing on the validation set to mitigate overfitting which is especially problematic when utilizing transfer learning of large models to smaller datasets (Srivastava et al., 2014). The ensemble classifier outputs a label for each individual image, corresponding to a 0.5-s window of the original audio clip. The output probabilities of all images corresponding to a given audio clip were summed cumulatively and averaged to give a resultant aggregate participant classification (Figure 1D). A fixed decision boundary of 0.5 was used to classify participants as either a fail (< 0.5) or a pass (≥ 0.5).

### Model training and testing

**FIGURE 3:**
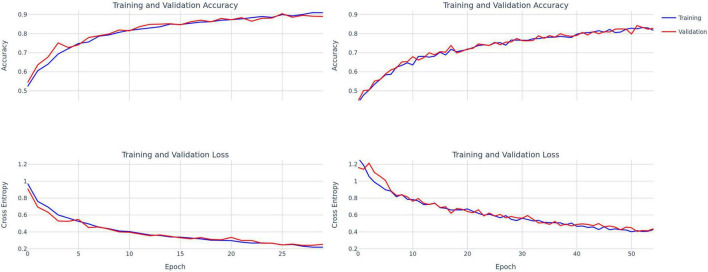
Training and validation accuracy and loss curves for ConvNext-Tiny **(left)** and DenseNet-121 **(right)**.

The first 40 participants’ vocal samples were selected for the training set (58.9% of total data) and the final 28 participants’ data (41.1% of the total data) for the test set. A testing split of 20% was used during the training phase. There was no overlap between training and testing sets. The decision to segregate data based on the order of participation rather than random sampling was deliberate. This choice aims to simulate a real-world scenario where the model is trained on existing data and is then required to generalize to new, unseen participants. Thirty and forty-five epochs were used to train ConvNext and DenseNet, respectively, with a learning rate of 1e-5 and a batch size of 32. A learning rate scheduler and early stopping were used to mitigate overfitting (Figure 3). Given differences in the number of parameters in each model, we used 55 and 30 target epochs with patience of 7 and 10 for DenseNet and ConvNext-Tiny, respectively, with validation loss as the early stopping parameter.

## Results

The mean age of the patients was 69 years. Most patients had ischemic stroke (76%), with 46% involving middle cerebral artery, 18% brainstem, and 1.5% thalamic infarcts. Others had approximately 9% multifocal infarcts, 3% cerebellar and 16% intracerebral hemorrhage (ICH). Within the training and testing cohort, demographics between TOR-BSST© pass and fails were compared (two-sample *t*-test for continuous data, Chi-squared test for frequency data). There were no differences between pass and fail patients in both cohorts except for higher NIHSS in patients who failed (Table 1); lower NIHSS was associated with a pass status as expected. Comparing training and testing cohorts there was no difference in baseline demographics (Supplementary Table 1) in a similar manner.

**TABLE 1:** Demographic characteristics of pass and fail patients within training and testing patient cohorts.

Training cohort			
	**Screening FAIL**	**Screening PASS**	***p*-value**
*N*	18	22	
Mean age, years (SD)	69 (17)	67 (16)	0.72
Female, *n* (%)	6 (38%)	12 (55%)	0.21
Mean NIHSS (SD)	9 (6)	4 (6)	0.010
Stroke type (%)			0.11
Ischemic MCA	44%	59%	
Ischemic lacunar	22%	14%	
Ischemic multifocal	6%	23%	
ICH	22%	0	
CVST	0	0	
Other	6%	4%	
**Testing cohort**			
*N*	10	18	
Mean age, years (SD)	73 (18)	65 (16)	0.28
Female, *n* (%)	3 (30%)	12 (67%)	0.19
Mean NIHSS (SD)	6 (6)	2 (1)	0.001
Stroke type (%)			0.65
Ischemic MCA	40%	44%	
Ischemic lacunar	40%	22%	
Ischemic multifocal	0	6%	
ICH	20%	22%	
CVST	0	6%	
Other	0	0	

Comparing within training and testing cohorts (two-sample *t*-test for continuous data, Chi-squared test for frequency data), there was no differences between pass and fail patients in both cohorts except for higher NIHSS in patients who failed. *P*-values for subtypes individually not computed as small subtype *n*-values. NIHSS, National Institute of Health Stroke Scale; CVST, cerebral venous sinus thrombosis; ICH, intracranial hemorrhage; MCA, middle cerebral artery.

**FIGURE 4:**
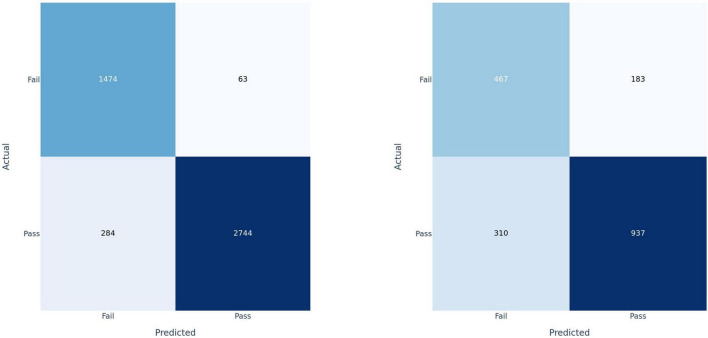
Confusion matrices for fusion model applied at the clip-level on training set **(left)** and test set **(right)**.

The training and test performance of the classifiers on both clip and participant levels are shown in Tables 2, 3. The performance metrics shown are sensitivity (recall), specificity, precision, F1 score, and area under the receiver operator curve (AUC). At the audio clip-level, DenseNet-121 demonstrated a sensitivity of 0.77, a specificity of 0.69, a precision of 0.56, an F1 score of 0.70, and an AUC of 0.79 [95% CI: 0.77, 0.81]. The ConvNext-Tiny model produced a sensitivity of 0.63, a specificity of 0.77, a precision of 0.58, an F1 score of 0.63, and an AUC of 0.78 [95% CI: 0.76, 0.80]. The ensemble fusion model, amalgamating DenseNet-121 and ConvNext-Tiny models, achieved a sensitivity of 0.71, a specificity of 0.77, a precision of 0.62, an F1 score of 0.73, and an AUC of 0.80 [95% CI: 0.78, 0.82] (see Supplementary Table 1 and Figure 4).

**TABLE 2:** Comparing RGB Mel-spectrogram and three-channel Mel-spectrogram clip-level classifier performance across DenseNet-121, ConvNext-Tiny, and a fusion of the two networks.

	RGB Mel-spectrogram					Three-channel Mel-spectrograms				
	**Sensitivity (recall)**	**Specificity**	**Precision**	**F1 score**	**AUC [95% CI]**	**Sensitivity (recall)**	**Specificity**	**Precision**	**F1 score**	**AUC [95% CI]**
DenseNet-121	0.78	0.69	0.56	0.70	0.79 [0.77, 0.81]	0.71	0.7	0.56	0.69	0.75 [0.73, 0.77]
ConvNext-Tiny	0.63	0.77	0.58	0.63	0.78 [0.76, 0.80]	0.66	0.74	0.57	0.69	0.76 [0.74, 0.78]
Fusion	0.71	0.77	0.62	0.73	0.80 [0.78, 0.82]	0.67	0.76	0.60	0.71	0.77 [0.75, 0.79]

CI, confidence interval.

**TABLE 3:** Comparing RGB Mel-spectrogram and three-channel Mel-spectrogram participant level classifier performance across DenseNet-121, ConvNext-Tiny, and a fusion of the two networks.

	RGB Mel-spectrogram					Three-channel Mel-spectrograms				
	**Sensitivity (recall)**	**Specificity**	**Precision**	**F1 score**	**AUC [95% CI]**	**Sensitivity (recall)**	**Specificity**	**Precision**	**F1 score**	**AUC [95% CI]**
DenseNet-121	0.89	0.79	0.67	0.81	0.89 [0.74, 1.04]	0.78	0.74	0.58	0.73	0.88 [0.73, 1.03]
ConvNext-Tiny	0.78	0.89	0.78	0.84	0.91 [0.77, 1.05]	0.78	0.79	0.64	0.77	0.86 [0.69, 1.03]
Fusion	0.89	0.79	0.67	0.81	0.91 [0.77, 1.05]	0.78	0.79	0.64	0.77	0.87 [0.71, 1.03]

CI, confidence interval, please note max. AUC value is 1.0, computed numerically has upper bounds larger than this value and is listed herein to show the calculation result.

Participants were classified based on the cumulative sum of output scores of each individual clip. At the participant level (Table 3), DenseNet-121 achieved a sensitivity of 0.89, a specificity of 0.79, a precision of 0.67, an F1 score of 0.81, and an AUC of 0.89 [95% CI: 0.74–1.04]. In contrast, the ConvNext-Tiny model delivered a sensitivity of 0.78, a specificity of 0.89, a precision of 0.78, an F1 score of 0.84, and an AUC of 0.911 [95% CI: 0.77–1.05]. Again, the fusion model showed a sensitivity of 0.89, a specificity of 0.79, a precision of 0.67, an F1 score of 0.81, and achieved the highest AUC of 0.912 [95% CI: 0.77–1.05] (Hanley and McNeil, 1982). We have included the third decimal place to show marginal difference between the two approaches. Confidence interval calculations are detailed in the Supplementary Methods section.

Using three-channel Mel-spectrograms instead produced slightly worse performance than RGB Mel-spectrograms, as detailed in Table 2 (clip-level performance) and Table 3 (patient-level performance). While the RGB representation offered certain benefits due to compatibility with transfer learning and standard CNN architectures, the overall advantage over the three-channel representation was observed to be marginal. Nonetheless, both methods produced comparable performance, indicating that either representation could be employed based on specific application needs.

Finally, the contribution of vowels to classification performance was also studied by comparing the results of vowels alone to vowels plus the speech components of the NIH. At the participant level, the additional information provided by the NIH speech components provided a significant increase in performance (Table 4).

**TABLE 4:** Participant-level performance using RGB Mel-spectrograms and only vowel sounds as inputs yielded reduced performance compared to using all recorded voice sounds.

	RGB Mel-spectrogram (vowels only)				
	**Sensitivity (recall)**	**Specificity**	**Precision**	**F1 score rage**	**AUC [95% CI]**
DenseNet-121	0.67	0.79	0.6	0.72	0.79 [0.44, 1.14]
ConvNext-Tiny	0.67	0.79	0.6	0.72	0.78 [0.39, 1.17]
Fusion	0.67	0.79	0.6	0.72	0.79 [0.42, 1.16]

CI, confidence interval, please note max. AUC value is 1.0, computed numerically has upper bounds larger than this value and is listed herein to show the calculation result.

## Discussion

The findings of our study support the use of deep learning, specifically convolutional neural networks employing transfer learning, as a tool for screening post-stroke dysphagia using real-world speech audio recordings. By leveraging established neural network architectures and ensemble methods, our approach achieved robust performance demonstrating its potential use as a non-invasive, time-efficient, and scalable screening tool in clinical settings. We hope that this tool can be used as an assistive technology (for example deployed on a mobile device), to aid any provider in performing a bedside swallow screening test. It also naturally lends itself to telehealth applications or other remote uses especially considering challenges introduced by the COVID-19 pandemic. Our proof-of-concept study supports the notion that this technology can be deployed in an assistive capacity to screen patients in low-resource settings constrained by person-power, off-hours access, or other challenges accessing screening services. Our models leveraged state-of-the-art CNN architectures (DenseNet-121 and ConvNext-Tiny), as well as a simple ensemble fusion approach to integrate the results of these architectures and improve classification performance. The fusion model’s results are promising, achieving a sensitivity of 0.89, specificity of 0.79, F1 score of 0.81, and an AUC of 0.91 when evaluated at the patient level, demonstrating compelling proof-of-concept results. This gain in performance is marginal relative to single models at the participant level, however, difference in AUC at the clip level suggests that the fusion approach would perform better when applied to a larger number of participants. Furthermore, the utilization of audio data, as opposed to other modalities allows for a less invasive collection method and integration into passive monitoring systems.

Prior approaches for classifying audio data predominantly employed classical statistical methods that necessitate the explicit extraction of signal features. While such methods are effective in specific scenarios, their application to voice-based dysphagia detection is constrained by the need for *a priori* knowledge of pathology-related acoustic features. These feature-centric methodologies have been utilized either in isolation or in conjunction with clinical variables for dysphagia identification (Roldan-Vasco et al., 2021; Park et al., 2022). Using a deep learning framework allows for reduced reliance on feature engineering and thus supports classification based on more complex signal characteristics using raw or minimally processed data. Some deep learning approaches have already demonstrated success in classifying speech into various classes such as identifying speakers by gender, accent, or other attributes (Khalifa et al., 2020; Wilhelm et al., 2020). Automatic dysphagia detection has also been studied using accelerometers or microphones attached directly to a patient’s neck to record swallowing sounds (Khalifa et al., 2020; O’Brien et al., 2021). Additionally, CNNs have been used extensively for audio classification using audio signals converted into images in fields outside of medicine (Hershey et al., 2017; Zhang et al., 2019; Palanisamy et al., 2020; Dave and Srivastava, 2023; Khurana et al., 2023).

Our deep learning approach produced results that are biologically plausible in that our model’s performance is within a range of reasonable expected values considering the limits of the studied population of patients. The underlying physiology of detecting dysphagia is quite complex, and thus no model or approach can be perfect for all populations. This is reflected in the current landscape of screening tests that have subjective interpretation and varied receiver-operator characteristics (Kiekens and Tognonato, 2022). With that said, our proof-of-concept study using deep learning suggests that with larger more diverse datasets this approach can converge to, or exceed, human operator performance with reduced subjectivity and variability. Our work, and indeed all established screening tests, are applicable only to patients with mild-to-moderate stroke as it requires a minimum awareness/consciousness and ability to follow some commands. In our case, the pass group expectantly had lower NIHSS scores.

Our study has several limitations, including a small dataset size, which can potentially introduce overfitting and limit the generalizability of our models. We did use real-world audio data gathered in clinical settings, which does improve certain aspects of generalizability and adoption by other centers; our code is also open source to facilitate wider use.^1^ We additionally attempted to address generalizability concerns by implementing robust model evaluation strategies, including early stopping during model training and using chronologically separated training and test datasets to mimic real-world multi-cohort testing. In future work, larger datasets, including non-English speakers, patients with accents, dental prosthesis, and other diverse populations will further expand generalizability.

Data-augmentation techniques in the audio domain such as time stretching, pitch shifting, or background noise injection could also be used to supplement our smaller dataset, however, this approach was not considered in this application given concerns about reduced explainability. However, the overlapping windows used to segment audio into spectrograms can be thought of as a type of cropping-based augmentation technique that maintains the integrity of the frequency-domain. Mel-spectrogram domain augmentation has also demonstrated promise in speech and acoustic scene classification and could be considered in future work (Wang et al., 2019). Additionally, input analysis should be explored in future work to determine the effects of varying recording conditions on model performance.

In this study we attempted to reduce the subjectivity involved with recognizing voice changes in dysphagia screening by developing a screening discriminator using TOR-BSST© “pass” or “fail” labels for the audio recordings as video fluoroscopy is not available for most patients. This is a limitation of our study, however, TOR-BSST© has been characterized as having excellent receiver operating characteristics that render it a good screening discriminator in a population assessed by VFSS, and in comparison, to many other screening tests (Kiekens and Tognonato, 2022). Our choice of labeling places this feasibility study as an assistive technology for care-providers and one that does not fully replace clinical judgment when coupled with bedside assessment (Liu et al., 2022). Furthermore, even in the setting of video assessment, there can be discordance between SLP reviewers reflecting the underlying physiological complexity of dysphagia.

Another consideration pertains to the manual segmentation of the audio data. Recognizing the potential scalability challenges associated with manual processing, we acknowledge the utility of automated segmentation techniques in streamlining the process. Nevertheless, the primary intent of this study was to establish the foundational feasibility of discriminating between dysphagia and non-dysphagia states via audio biomarkers. The manual approach was adopted considering challenges encountered in our real-world data collection environment (in hospital) and the occasional capture of the study data collector’s voice in the recordings. Moving forward, as this approach is further refined, automated segmentation with its promise for increased scalability will certainly be an area of focus and exploration.

Although screening tools generally exhibit good sensitivity and specificity, stroke patients commonly have a non-linear clinical course that can result in a fluctuating swallowing status. We utilized the most up-to-date swallowing screening result as a label for our data. This property is inherent to stroke physiology (at least in mild to moderate stroke), as there is a degree of spontaneous improvement over the course of several days. We attempted to mitigate this by assessing patients early in their clinical course. In the future, rapid ML-based clinical screening tools may allow fast serial assessments of dysphagia evolution and not simply one-time snapshots.

A further limitation we recognize are the challenges associated with applying CNNs, originally trained on image datasets, to spectrograms. This is due to the fundamental differences between these two types of data. Unlike images, spectrograms operate within unique parameter spaces, characterized by axes of frequency, time, and power. Furthermore, the non-local spectral properties of sound, and the inherent temporal nature of sound as noted by Hershey et al. (2017), Zhang et al. (2019), Palanisamy et al. (2020), Park et al. (2022), Dave and Srivastava (2023), add to this limitation. Nonetheless, CNNs are extensively used for analysis of audio signals and our findings and AUC measurements are congruent with these known limitations. We recognize the inherent variability and limitations that exist with real-world patient data, CNNs, and their ability to classify a physiologically complex pathology such as dysphagia.

## Conclusion

Our study demonstrates the feasibility of deep learning as an effective application for the screening of post-stroke dysphagia from vocalizations alone. This approach offers an avenue for the development of future non-invasive, less subjective, and rapid screening tools for dysphagia. This could contribute to improved patient management, outcomes, and democratization of swallowing screening.

## Data availability statement

The datasets presented in this article are not readily available because REB at Sunnybrook Health Sciences requires human voice data to be stored and analyzed locally. Requests to access the datasets should be directed to h.khosravani@utoronto.ca.

## Ethics statement

The studies involving humans were approved by Lisa Di Prospero, Sunnybrook Hospital, REB. The studies were conducted in accordance with the local legislation and institutional requirements. The participants provided their written informed consent to participate in this study.

## Author contributions

RS: Conceptualization, Data curation, Formal analysis, Investigation, Methodology, Project administration, Software, Visualization, Writing – original draft, Writing – review and editing. AB: Conceptualization, Methodology, Data curation, Investigation, Writing – original draft, Writing – review and editing. HM: Methodology, Software, Writing – review and editing. EN: Methodology, Writing – review and editing. LP: Methodology, Writing – review and editing. AW: Writing – review and editing. AS: Writing – review and editing. GR: Writing – review and editing. MC: Writing – review and editing. MB: Writing – review and editing. BM: Writing – review and editing. HK: Conceptualization, Methodology, Data curation, Investigation, Resources, Writing – original draft, Writing – review and editing, Supervision, Project administration, Funding acquisition.

## Footnotes

1
https://github.com/UofTNeurology/masa-open-source
