# Supplementary material for: Machine-learning assisted swallowing assessment: a deep learning-based quality improvement tool to screen for post-stroke dysphagia
Source: Front Neurosci. 2023 Nov 24;17:1302132. doi: 10.3389/fnins.2023.1302132 (PMC10734030; doi:10.3389/fnins.2023.1302132)
Supplement: Supplementary file 1 [file Data_Sheet_1.docx]

**Supplementary Section**

**Supplementary Methods:**

**Data Analysis Codes:**

Python codes used for all data analysis and machine learning can be found at the following link: https://github.com/UofTNeurology/masa-open-source

**Data Collection:**

Noise was minimized by recording patients with all IV alarms off temporarily once determined to be safe and either in a one-person room, or with nearby patients requested to try not to speak. If an over-head hospital page occurred, the recording was redone (for the segment affected).

**Data Quality Assessment:**

Three raters (RS,AB,HM) underwent a calibration process to ensure consistency in their ratings. This involved a training session where they collectively reviewed and rated a set of sample audio clips, discussing and resolving any discrepancies in their assessments.

The raters were tasked with evaluating the voice clips on several specific parameters, including subjective loudness of the vocal sample, presence of background noise, and clarity. Each parameter was rated on a scale from 1 (poor) to 5 (excellent), and the scores were then averaged to provide a composite voice quality score for each clip. The raters were blind to each other's scores and to any clinical information about the patients. Each rater evaluated the clips at their own pace, with the ability to replay clips as needed for accurate assessment. Discrepancies in scores were resolved through discussion until a consensus was reached. After this process, only 1 participant was rejected for poor audio data quality.

**Mel-spectrogram Image Types**

Two types of Mel-spectrogram images were used to train machine learning classifiers. The first (RGB Mel-spectrogram) used red-blue-green (RGB) images of Mel-spectrograms directly as inputs to the CNN. This approach has the advantage of reduced number of computations required to produce a resultant image and produces an input image that is more similar source and target domains when using a CNN pre-trained using ImageNet weights. The second approach (termed “Three-channel Mel-spectrogram”) involves the depth-wise concatenations of three-monochrome Mel-spectrograms and has demonstrated superior performance compared to RGB images in some applications (32). In this alternative approach each RGB channel is replaced by a Mel-spectrogram with a different FFT window size. This produces a single image that can contains multiple time-frequency resolutions. We used the following window sizes: 1024, 2048, and 4096 corresponding to window time lengths of approximately 21, 42 and 84 ms respectively. In our case, we maintained the same hop length (64) across FFT windows to produce a consistent output array size and to facilitate array concatenation.

**Convolutional Neural Network Architecture:**

For both DenseNet and Convnext, the network weights were initiated to pre-trained ImageNet weights, the top layer of the neural network was removed and replaced by a 2D global average pooling layer and an output dense layer as the final prediction layer. To mitigate against overfitting a drop-out layer was added before prediction output with a dropout rate of 80%. Early stopping was also employed during model training to further mitigate against overfitting. A base learning rate of 1e-4 with an exponential decay learning rate scheduler, with a batch size of 32, was used to train each model independently on the training dataset. Given the relatively small dataset a small number of epochs were used to train each network (35 for Convnext, and 50 for Densenet). The ensemble classifier output produced a label for each individual image, corresponding to a 0.5-second window of the original audio clip. The output probabilities of all images corresponding to a given audio clip were summed cumulatively to give a resultant aggregate participant classification. A fixed decision boundary of 0.5 was used to classify participants as either a fail (< 0.5) or a pass (>= 0.5). For AUC CI calculations we used the standard equations as outlined in [31].

**Supplementary Table 1:** Demographic characteristics of training and testing patient cohorts compared overall.

|  | Training Cohort | Testing Cohort | *p*-value |
| --- | --- | --- | --- |
| N | 40 | 28 |  |
| Mean age, years (SD) | 70 (16) | 66 (16) | 0.99 |
| Female, n (%) | 19 (48%) | 14 (50%) | 0.32 |
| Mean NIHSS (SD) | 6 (6) | 4 (4) | 0.018 |
| Stroke type (%) |  |  |  |
| Ischemic MCA | 53 | 46 |  |
| Ischemic Lacunar | 12 | 29 |  |
| Ischemic Multifocal | 15 | 0 |  |
| ICH | 12 | 21 |  |
| CVST | 0 | 4 |  |
| Other | 8 | 0 |  |

Abbreviations: NIHSS = National Institute of Health Stroke Scale, CVST = Cerebral Venous Sinus Thrombosis, ICH = Intracranial Hemorrhage, MCA = Middle Cerebral Artery
